# Student Education Management Strategy Based on Artificial Intelligence Information Model under the Support of 5G Wireless Network

**DOI:** 10.1155/2022/4709146

**Published:** 2022-07-01

**Authors:** Yushu Guan

**Affiliations:** Fine Arts College, Shenyang University, Shenyang 110044, China

## Abstract

With the popularity of the Internet and the advancement of information technology, more and more people are accepting the teaching and sharing of knowledge through the digitalization of information. The widespread adoption of 5G technology has pushed online learning even further into the mainstream. However, because online teaching does not have the drawback of being intuitive like classroom teaching, teachers' assessments of students' learning situations are less accurate. As a result, how to effectively evaluate students' academic performance in the context of 5G wireless network technology is a pressing issue that must be investigated. By processing these heterogeneous large-scale learning records and integrating multiple perspectives to analyze this learning record information to identify students' learning behaviors, this study proposes an integrated analysis algorithm based on artificial intelligence information technology. The possible learning outcomes of students are predicted based on their current learning situation, so teachers can provide auxiliary teaching strategies to students who may have learning difficulties based on the predicted information. The method proposed in this article uses information technology to predict students' grades, and the analysis shows that the method is very effective. In this article, different grades of classification methods are used to analyze and predict the whole students. All grade classification methods are effective in describing decision rules. No matter what grades classification method is used, the error rate of students' grades distribution is predicted to be below 40%.

## 1. Introduction

5G networks have significant advantages over traditional 3G and 4G communication networks, including high information transmission efficiency and relatively fast speeds. It is organically combined with big data, the Internet of Things, and other technologies, greatly promoting business needs and business model innovation in a variety of fields and providing strong support for society's intelligent development. Artificial intelligence is formed when computer technology and communication technology are combined in this way. Massive amounts of data are generated every second in the big data environment [1]. Artificial intelligence can greatly improve the speed and quality of data processing and be used to process large amounts of complex data, increasing data security. Artificial intelligence, when used extensively, not only improves the information processing capability of computer systems but also promotes their evolution toward intelligence and automation. Furthermore, it can completely ensure the system's stability. Massive amounts of data are generated every second in the big data environment. Artificial intelligence can greatly improve the speed and quality of data processing and be used to process large amounts of complex data to improve data security [2]. With the development of artificial intelligence and the arrival of the 5G communication era, in order to realize the effective management of massive network information, advanced artificial intelligence technology can be adopted, many scientific and reasonable solutions can be compiled, and an expert knowledge sharing system can be established to gradually improve the data analysis and processing effect. On the other hand, experts in the field of theory should effectively strengthen scientific research, summarize theoretical results, and fully reflect the advantages of artificial intelligence.

Based on artificial intelligence information technology, this article proposes an algorithm for predicting students' academic performance. The decision tree's input and output variables are determined by the algorithm using the student learning behavior factor data table [3]. Then, all samples are randomly divided into three datasets, which are used for training, testing, and validation, and the characteristics of students' learning behavior are analyzed accordingly, resulting in an algorithmic basis for predicting students' grades. Find the decision-making rules between students' various learning behavior attributes and learning outcomes for each course in the fifth and sixth semesters and explain the results. This study uses the same course in different semesters as the object of verification, and the data from the fifth, sixth, and seventh semesters as training data and test data, respectively, to verify the discovered decision rule. The training error rate and the test error rate are used as verification indicators to try to find the best prediction time point using different time units and to try to find the best grade classification method using various grade classification methods. The artificial intelligence-based decision tree for predicting students' academic performance analyses and discusses the students' learning situation and provides specific educational guidance based on the data analysis results [4].

This study examines the relationship between course attributes and decision rules under different courses, the relationship between different time points and decision rules under different time units, and the relationship between different grade classification methods and decision rules by processing these heterogeneous large-scale learning records, the relationship between each time point within the time unit and the high and low score prediction effect, the relationship between different grade classification methods and the high and low score prediction effect, and so on. Students' current learning circumstances predict their potential learning effect [5].

This article's chapter structure is as follows: this article extracts data from learning records, uses artificial intelligence technology to classify and analyze the data, and then provides teachers with information on the relationship between students' learning behavior and learning effectiveness, as well as the degree of correlation between the two. Teachers can predict various learning situations that may exist in current and future students based on their past learning behavior and provide learning assistance or adjust teaching strategies in real time.

The following are the article's unique features: the first chapter introduces related research on artificial intelligence information technology and teaching strategies; the second chapter examines current educational management needs in light of current educational development; the third chapter is based on artificial intelligence information technology. The fourth chapter uses intelligent information technology to quantitatively analyze the results and summarize the rules, and the fifth chapter is a summary of the entire text.

## 2. Related Work

The international empirical analysis of student achievement prediction has also developed from the original manual statistics to the joint statistics of artificial intelligence and information technology.

Combined with the current situation of information technology teaching in junior high schools, Kaptein MC built a junior high school information technology teaching model with the concept of flipped classroom teaching and proposes to improve learners' interest in information technology through independent learning or cooperative exploration among learners and cultivate students' ability in the learning process. In terms of research on learning motivation, creativity, and initiative [6], Zhou H tried to revise and perfect the new model in teaching practice through three rounds of action research. Through questionnaire survey and SOLO taxonomy, the impact of this model on the deep learning and learning ability of primary school students was analyzed [7]. After in-depth research, Luo X built a grade prediction model. By using this model, the characteristics of students can be predicted, and then the low-achieving students can be analyzed in a targeted manner [8]. Huang X collected students' learning information through questionnaires, studies students' learning behavior and then converted students' tacit knowledge into explicit information through the Apriori algorithm and cluster analysis, and this explicit information can be used as follow-up services so as to improve students' learning satisfaction [9]. Kirichenko A V et al. used a combination of a self-organizing competitive neural network (SOM) and BP neural network to establish a student achievement prediction model [10]. After intensive research, Cui L proposed a system that can predict student grades based on multiagent learning (MAFDS) behavior [11]. Jie H used two data mining methods, genetic algorithm and decision tree, to establish a model to find out the potential relationship between factors in student behavior data and use a decision tree for preliminary classification [12]. Zhang Z used three methods: support vector machine, logistic regression, and decision tree C5.0 to construct a low learning achievement risk classification model, and they used a 10-fold cross-validation method to verify the model and found that the method with the best prediction effect was support vector machine [13] ]. Ting-Ting LU used a variety of data mining methods to integrate expert opinions and then used genetic algorithms to find the best combination solution to create an optimal integrated student achievement prediction model. The best prediction method is a neural network [14]. Liu Y's research found that the classification and prediction of students' grades is an important problem, and the commonly used classification methods often include the logistic regression model and Bayesian algorithm. Chen Y used the decision tree in data mining technology to classify the data mining application of predicting students' grades. The results show that the expected evaluation effect can be achieved by using the decision tree [16]. Zhang X pointed out that the use of support vector machines has achieved good prediction results in terms of student achievement, and studies have shown that the application of support vector machines in feature selection and prediction has achieved high classification accuracy [17].

In literature, it is generally emphasized that the application of innovation theory is used to reform teaching methods, but there are no articles that specifically propose methods and strategies for various management work in colleges and universities, such as teaching management, student management, campus culture construction, and enrollment work. Through the research on the application of data mining in student achievement prediction, it can be found that at present, most of the research on student achievement prediction is focused on students' examination results, but there is no research on how to effectively predict students' academic achievements under the new teaching method such as online education, and an in-depth study on the correlation between academic achievements and learning courses, learning time and learning patterns.

## 3. Education Management Needs Analysis

Most of the talents cultivated by higher education will become leaders and leaders of all walks of life and can be regarded as high-end talents in all walks of life. For this type of talent, in the actual leadership work, they need to put aside traditional and conservative old views and old ideas, so they need a more innovative spirit. As a cutting edge of the education industry, higher education should pay more attention to the cultivation of innovation ability and apply the innovation concept to daily education management. Since the concept of artificial intelligence information technology was put forward, more and more people began to realize the importance of innovation, and more and more people began to put it into practice [18]. Nowadays, the trend of educational development is no longer to align with scores, no longer to train high flyers of top universities, and no longer to blindly ask students how many scores they get in TOEFL and IELTS but to pay more attention to students' personal ability while valuing scores. Students with high test scores, low abilities, and a lack of innovative ability are already unpopular, and in today's information age, they will be questioned and eliminated. They are energetic, persistent, focused, imaginative, and adventurous; they have a strong ability to learn and explore themselves; they have broad and solid knowledge, a high professional level, good moral cultivation, and the ability to cooperate or coexist with others. True innovative talents with the aforementioned characteristics will become the most in-demand talents in today's society and Muhui's mainstay. Artificial intelligence information technology, on the surface, refers to making improvements to original educational concepts, educational methods, and educational methods in order to make them more current and effective. In essence, artificial intelligence information technology is used to break through the ingenious vertebrae of previous educational development through educational innovation, making education more grounded and in line with the needs of the times, rather than simply activating the classroom atmosphere and enriching the content of textbooks. In the new situation, education reform is focusing on improving students' innovative awareness and ability [19, 20].

**Figure 1:**
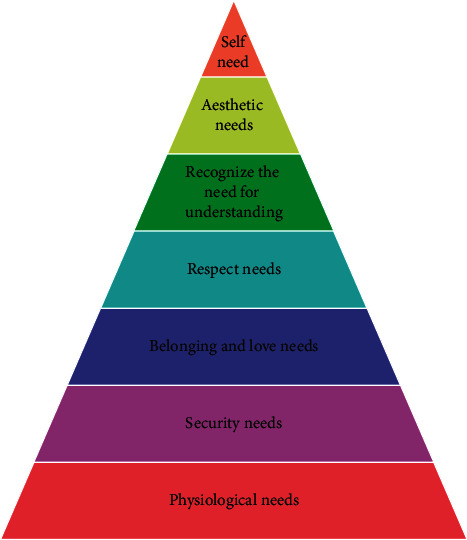
Need hierarchy theory diagram.

Constructivist learning theory believes that learning is the process of students actively constructing internal mental representations in the process of interacting with the learning environment. Knowledge is not obtained through the teacher's teaching and transmission but is obtained by students in a certain situation, with the help of other means, using necessary learning materials and learning resources, and by means of meaning construction. Constructivism puts forward the viewpoint of situational cognition, emphasizes the situational nature of learning, knowledge, and wisdom and believes that knowledge cannot exist abstractly without being separated from the activity situation, and learning should be combined with situational social practice activities. These need to be arranged in a hierarchy from low level to high level, as shown in Figure 1.

The four needs at the bottom of the hierarchy are classified as missing needs, which are essential for individual survival and must be met to some extent [21]. When all of the missing needs are met, the individual will pursue the above three high-level needs, which are classified as growth needs and can improve the quality of the individual's life. According to Maslow's hierarchy of needs theory, everyone has a need for understanding, respect, and self-realization, and everyone has a desire and need to communicate. Even the most withdrawn, independent person, or the so-called poor students in the eyes of some teachers, hope to receive understanding, care, and assistance in their hearts. Students can benefit from rich learning resources, convenient interaction methods, and a humanized situation design in the information technology teaching environment, which can meet their communication, respect, knowledge, and aesthetic needs. Once these needs are met, students will have a positive attitude toward themselves and gain positive emotional experiences like satisfaction, happiness, and pride, which will aid in developing advanced social emotions like reason, beauty, and morality so that the students have a positive mental attitude [22].

## 4. Quantitative Evaluation of Teaching Based on Artificial Intelligence Information Technology

The ultimate goal of this article is to improve the level of school teaching management, make teaching management more convenient and fast in the future, digitize teaching evaluation information, and allow users of teaching evaluation to access the system from any location and time on the campus network without being limited by time and space. The purpose of reviewing the information you need is diversified, which can meet the current practical needs of various evaluation purposes. At the same time, focusing on the fundamental purpose of promoting the development of teachers, the main functions of the teaching management unit are transferred to the collection, processing, and transformation of digitalization, the realization of technical means, and the transformation of service methods [23]. After an in-depth analysis of the problems, when the factors contained in the problems are divided into different levels, such as target level, criterion level, index level, scheme level, and measure level, the subordinate relationship of hierarchical structural factors of the levels is explained in the form of the block diagram. When a certain level contains many factors, the level can be further divided into several levels. Since it is quite difficult to directly use the definition to calculate the eigenroot and eigenvector of the matrix, after the paired comparison matrix is constructed, the sum-product method or the square root method can be used to approximate the maximum eigenroot and corresponding weight vector of the constructed paired comparison matrix. Using the sum-product method to calculate, the formula is shown in as follows:

(1) $$\begin{array}{c} a_{ij}=\frac{a_{ij}}{\sum_{k=1}^{n}a_{kj}}\left(i=1,2,\ldots ,n\right). \end{array}$$

For the judgment matrix normalized by column, sum it by row as shown in the following:

(2) $$\begin{array}{c} {\bar{\omega }}_{i}=\sum_{j=1}^{n}{\bar{a}}_{ij}. \end{array}$$

Normalize the vector $\bar{\omega }={\left[{\bar{\omega }}_{1},{\bar{\omega }}_{2},\ldots ,{\bar{\omega }}_{n}\right]}^{T}$ as shown in the following:

(3) $$\begin{array}{c} \omega_{i}=\frac{{\bar{\omega }}_{i}}{\sum_{i=1}^{n}{\bar{\omega }}_{i}}. \end{array}$$

In the actual evaluation, the evaluation experts can only roughly estimate the value of the element *a*_*ij*_ in the pairwise comparison matrix, which often leads to inconsistent logic errors. In this article, the difference between *λ*_max_ and *n* is used to test the consistency. First, calculate the maximum eigenvalue such as the following equation:

(4) $$\begin{array}{c} \lambda_{\max}=\sum_{i=1}^{n}\frac{{\left(A\omega \right)}_{i}}{n\omega_{i}}. \end{array}$$

Calculate the consistency index as shown in the following equations:

(5) $$\begin{array}{c} CI=\frac{\lambda_{\max}-n}{n-1}, \end{array}$$

(6) $$\begin{array}{c} CR=\frac{CI}{RI}. \end{array}$$

When *CR* < 0.1, the inconsistency scale of task A is within the allowable range. Otherwise, the evaluation expert needs to reestimate and adjust the value of the element *a*_*ij*_ in the pairwise comparison array until the consistency requirements are met. The average consistency principle is shown in Table 1.

**Figure 2:**
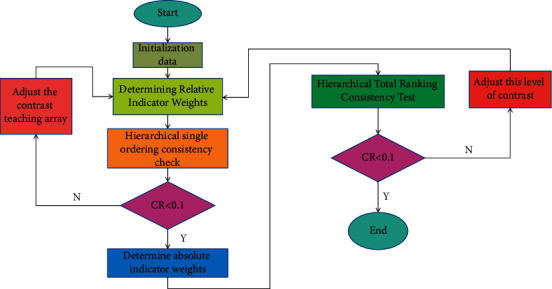
Flowchart of weight indicator.

After obtaining the relative weights between the indicators of the same layer, the absolute weights of the indicators at all levels can be calculated from the top to the bottom. Overall consistency is considered acceptable when *CR* < 0.1. Otherwise, it is necessary to adjust this paired comparison matrix until the consistency test of the total ranking of the hierarchy meets the requirements. In summary, the process of calculating the absolute weight can be obtained as shown in Figure 2.

Since the indicators have different unit dimensions, the indicators must be standardized, and all indicators are converted into indicators without units. There are positive indicators, reverse indicators, and moderate indicators in the indicator system, and the advantages and disadvantages of the indicators are not clear. Therefore, the method of fuzzy quantization, that is, fuzzy membership function, is used to carry out dimensionless processing of each indicator [24].

For the positive index, the semirising trapezoidal fuzzy membership function is used to quantify, and the processing of the positive index is shown in the following:

(7) $$\begin{array}{c} x_{ij}=\frac{x_{ij}-x_{\min}}{x_{\max}-x_{\min}}. \end{array}$$

For the reverse index, the semidrop trapezoidal fuzzy membership function is used to quantify, and the reverse index processing is shown in the following:

(8) $$\begin{array}{c} x_{ij}=\frac{x_{\max}-x_{ij}}{x_{\max}-x_{\min}}. \end{array}$$

For the moderate index, the semisublimation descending trapezoidal fuzzy membership function is used to quantify the moderate index processing, as shown in the following:

(9) $$\begin{array}{c} x_{ij}=\left\{\begin{array}{c} \frac{x_{ij}-x_{\min}}{x_{half}-x_{\min}},x_{ij}>x_{half},\\ 1,x_{ij}=x_{half},\\ \frac{x_{ij}-x_{\min}}{x_{\max}-x_{half}},x_{ij}<x_{half}. \end{array}\right.. \end{array}$$

The decision-making rules generated by artificial intelligence information technology analysis are for each course, and most of the analyzed data are historical data of the entire semester. For teachers, these decision-making rules are not directly helpful to the courses being taught, and they are based on the data of the whole semester. Whether these decision-making rules can be applied to the same courses in different semesters is indeed necessary to verify the accuracy of these decision-making rules in predicting the courses.

## 5. Data Analysis

### 5.1. Test Score Indicators

The median is the value of a point in the center to represent the central tendency of a set of data. The median is suitable not only for data with skewed distribution, irregular distribution, large difference between variable values, and unbounded at one or both ends but also for data with symmetrical distribution or unclear distribution. The arithmetic square root value of each variable value from the square of the mean deviation, also known as the square root of deviation from the mean. As an overall parameter, it is usually represented by a symbol, and the formula is shown as follows:

(10) $$\begin{array}{c} \sigma =\sqrt{\frac{{\sum \left(X-u\right)}^{2}}{N}}. \end{array}$$

**Figure 3:**
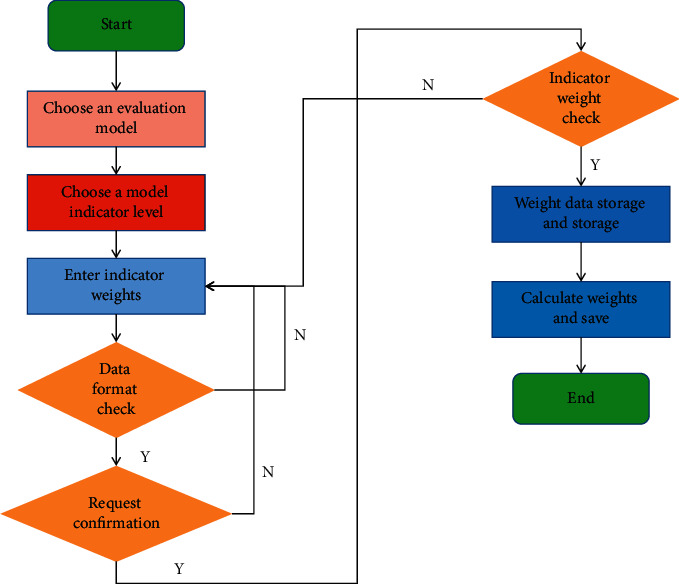
The flowchart of test paper evaluation model verification.

Evaluate the overall difficulty and discrimination of the test paper, and conduct difficulty analysis and discrimination analysis for each question. Through analysis, we can better understand the degree of difficulty and distinction of the test paper and evaluate the quality of the test paper as a whole. Evaluate the option ratio distribution of multiple-choice questions and design questions more scientifically. The test paper evaluation model validation process is shown in Figure 3.

Since the records of the log files are too scattered, they have no substantial meaning and help for teachers or students. Therefore, it is necessary to combine the basic data files of students and the data of the course database of the relationship between course information and students. After preprocessing and after the data are sorted and processed, a learning record database describing the learning behavior of each student in each course can be further established, which can be used as a reference for quickly querying the real-time learning status of students in each course in the future.

### 5.2. Data Analysis of Student Achievement Prediction

Analyzing the decision-making rules that link students' learning behaviors and learning outcomes in each course can help teachers and teaching assistants understand what learning behaviors students exhibit in this course and how they may affect academic performance. They can also observe the attribute value changes of related learning behaviors in subsequent lessons and provide assistance ahead of time. However, it is understandable to analyze these learning behaviors using only data from the entire semester; however, these decision rules can only describe students' learning behavior during the previous entire semester of the course, not at different time points, because the real learning behavior is not predictable. We can predict the possible learning effects of students based on their current learning situation and give the purpose of auxiliary teaching in advance by observing these learning behaviors. This study uses the training error rate and the prediction error rate as comparison indicators for various analyses when validating the decision rules in order to verify the relationship between each time point and the decision rules. The goal of tracking the training error rate is to avoid having a high training error rate. The prediction error rate refers to the error rate of the decision rules trained in the sixth semester as the test data in the seventh semester. It can be used to ensure that decision rules are applied consistently across semesters. As a result, the focus of this section will be on observing the relationship between each time unit and the prediction error rate. It is primarily based on the cumulative amount of learning behavior attribute values at various time points; that is, the cumulative value of each frequency is calculated from the start of the school day to each time point, and by observing the relationship between changes in the cumulative amount of learning behavior variables at various times and the learning effect, to provide future decision tree analysis, by observing the relationship between attributes and the learning effect. The cut-off points for grades in this article are 10 points, 20 points, 25 points, and 33 points, and the time variables used in most previous studies are used as the observation dimensions of learning type analysis. In other words, whether the attributes in the decision rules are the same at all time points and whether it is possible to predict ahead of time which students will fall into the low-scoring group and have poor learning outcomes before the semester ends and provide remedial teaching or a related teaching strategy. Students' final semester grades in the course are used to assess learning outcomes.

**Figure 4:**
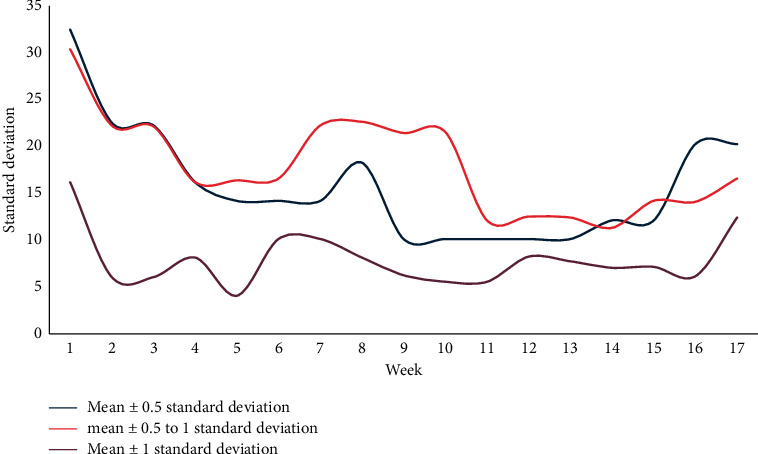
The standard deviation of the training error rate at each time point with 7 days as the time unit.

**Figure 5:**
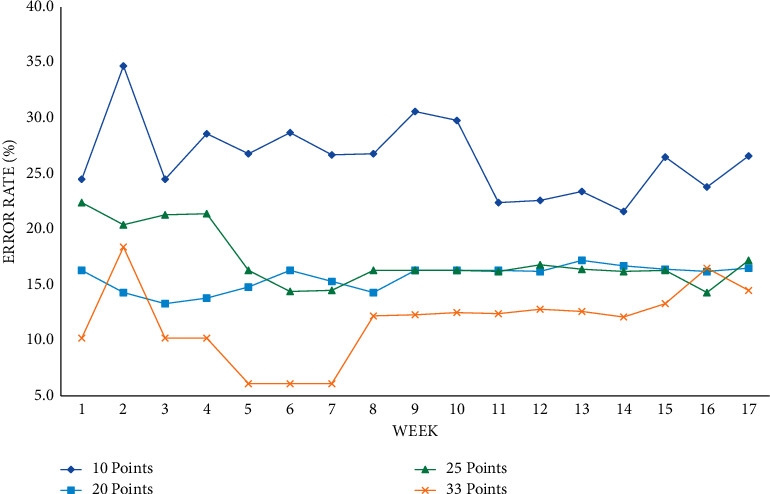
Training error rate at each time point with 7 days as the time unit.

This article first observes the changes and trends of the training error rate and prediction error rate at various time points under different time units such as seven days, fourteen days, and twenty-eight days. Finally, a conclusion is drawn by comparing the prediction effects of different time units. The training error rate at each time point with seven days as the time unit is shown in Figures 4 and 5.

Taking seven days as a time unit, the training error rate of various performance classification methods can be below 30%, and the average error rate of the mean ± 1 standard deviation can be trained in the eighth to ninth weeks. The performance is lower if it falls below 15%, and each classification method can reduce the error rate over time and drop below 20% in the score range of 20, 25, and 33 points.

**Figure 6:**
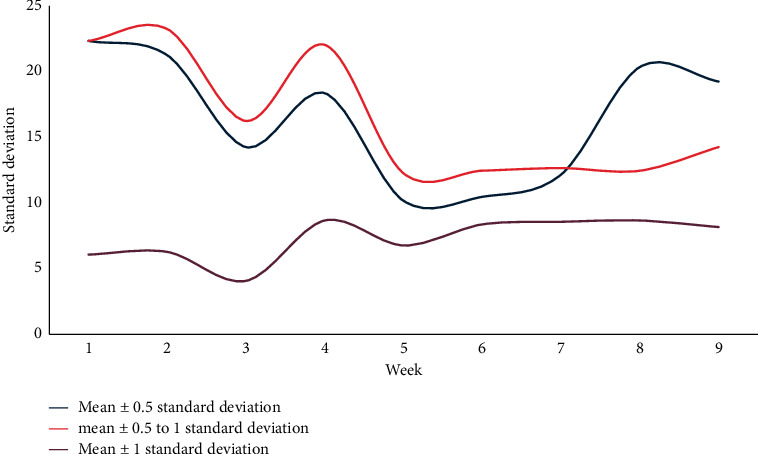
The standard deviation of the training error rate at each time point with fourteen days as the time unit.

**Figure 7:**
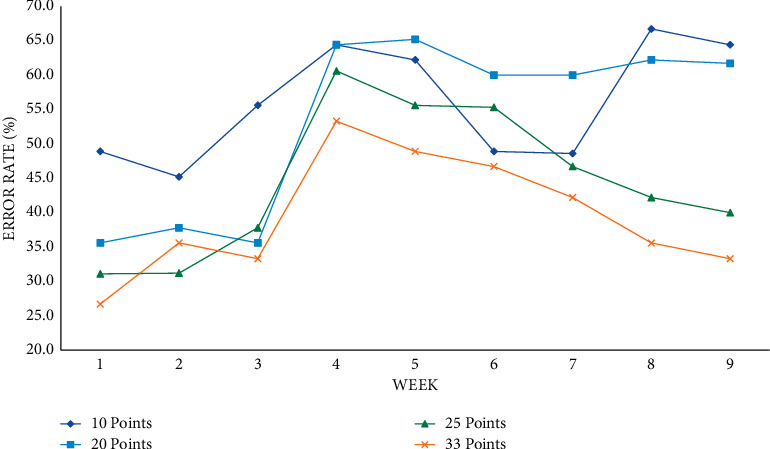
The training error rate at each time point with fourteen days as the time unit.

The training error rate at each time point with 14 days as the time unit is shown in Figures 6 and 7.

Figure 7 shows the prediction error rate at each time point under the time unit of fourteen days. Taking 14 days as the time unit, the prediction and practice error rates of various performance classification methods can be below 50%, and the average performance of the error rate with the mean ± 1 standard deviation is lower and the mean ± 1 standard deviation can drop to the error rate below 35% in weeks seven to eight. Among them, if compared with Figure 7, it can be seen that the two methods of mean ± 0.5 to 1 standard deviation and 20 points are the two methods, the prediction effect in the time unit of 14 days has become less obvious, so it is concluded. Therefore, as the time period increases, the effect of reducing the error rate will become less obvious, and the two are inversely proportional.

**Figure 8:**
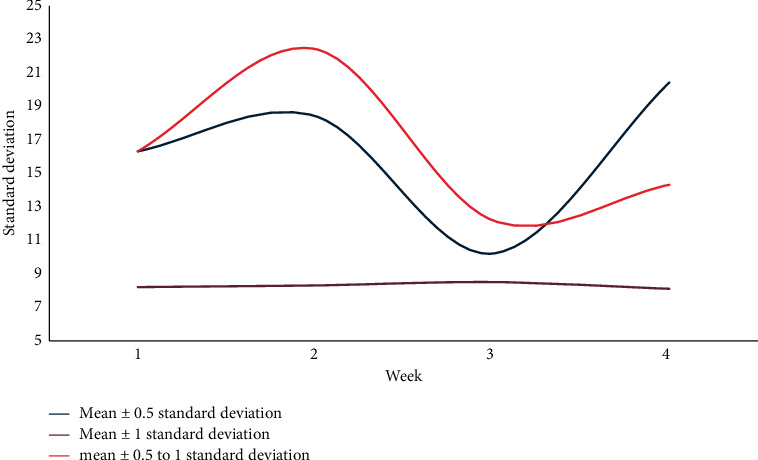
The standard deviation of the training error rate at each time point with twenty-eight days as the time unit.

**Figure 9:**
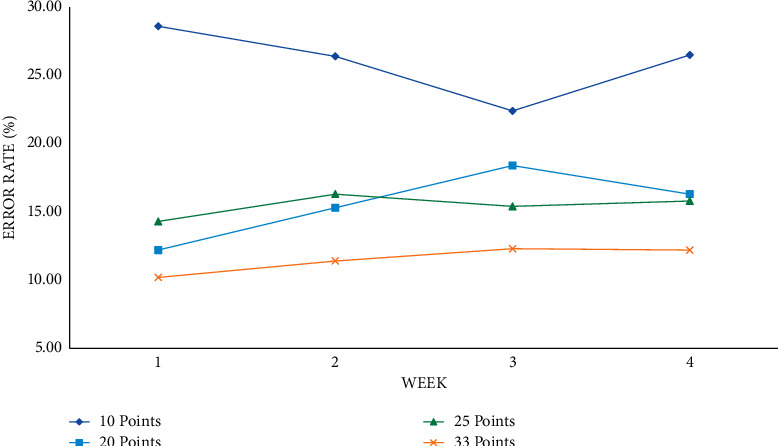
The training error rate at each time point with twenty-eight days as the time unit.

The training error rate at each time point with twenty-eight days as the time unit is shown in Figure 8 and Figure 9.

Figure 9 shows the training error rate at each time point under the time unit of twenty-eight days. Taking twenty-eight days as the time unit, the mean ± 1 standard deviation, and the difference between the mean ± 0.5 and 1 standard deviation, the average performance for error rates is low, all below 40%. As mentioned earlier, the relationship between the accumulation over time and the decrease in error rate becomes less and less obvious, and the way of representing the time forecast in months is not ideal.

### 5.3. Prediction Error Rate Analysis of Different Grade Classification Methods

**Figure 10:**
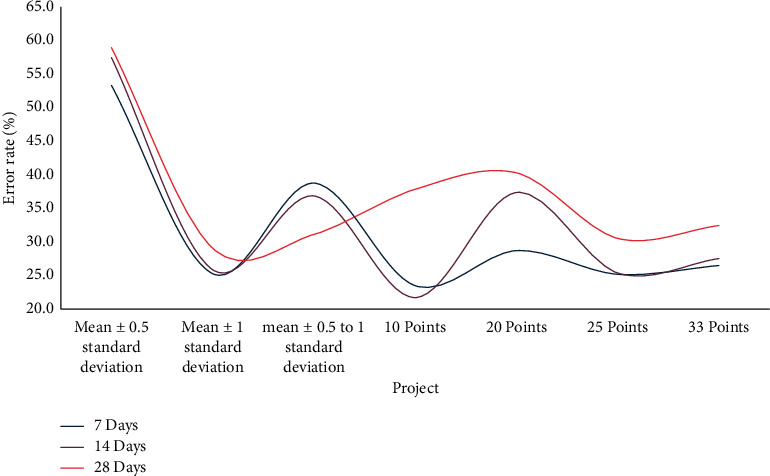
Prediction error rate at each time point in different time units.

The decision-making rules of learning behavior and learning effect of various time units and different grades can be predicted for all students taking the course under different time units, but these decision-making rules are used regardless of the type of grades used. The accuracy of predicting a student's overall learning behavior and learning effect is not very good. Each time point under different time units will be used as the analysis point of observation and prediction in this subsection. The average training error rate in different time units for each time point: the prediction error rate at each time point under different time units is shown in Figure 10.

From Figure 10, an interesting phenomenon is found for the prediction error rates of high-segmentation groups at various time points in different time units; that is, from the perspective of seven days as a time unit, each classification method is the error rate at the beginning. The lowest is the lowest and then gradually increases. The effect of prediction in different time units is not very different. However, different classification methods of grades have a strong relationship with the effect of prediction for high-scoring groups. In this article, different grade classification methods are used to analyze and predict the whole students. All grade classification methods are effective in describing decision rules. No matter what grades classification method is used, the error rate of students' grades distribution is predicted to be below 40%. Other predictions are good, and it can be seen that in terms of average predictions, the results analyzed in this study can indeed effectively predict the distribution of academic performance.

To summarize, the longer the time unit, the less obvious the effect of lowering the error rate. High-scoring students' learning behavior, on the other hand, is more difficult to predict. The error rate starts out low and gradually rises. The effect of various grade classification methods on the prediction will then be examined. Whether it is seven days or seven months, the prediction effect is the same. Most grade classification methods can be significantly reduced after the eighth week, whether in terms of time units of 14 days or 28 days. This has more meaning for teachers because it means that the students' learning status and learning behavior can be observed in real time during the semester using the method used in this study, and which students may fall into the low-scoring group can be predicted in advance using the method used in this study. Students with low learning effectiveness are identified so that teachers or teaching assistants can provide immediate assistance in order to achieve targeted programme teaching.

## 6. Conclusions

The goal of this study is to develop an integrated analysis algorithm that will provide teachers with decision-making rules as auxiliary information for teaching, allowing them to instantly understand students' learning status during class and provide different teaching or assistance to students with different learning behaviors. It can help teachers and teaching assistants understand what kind of learning behavior students perform in the course, which may lead to a final academic performance of a high or low score, and can observe the attribute value changes of related learning behaviors when the same course is offered again, using intelligent information technology to analyze the decision-making rules between students' learning behavior and learning effectiveness in each course. This study uses the same course in different semesters as the verification object, and the data from two semesters as training and test data, respectively, and the training and test error rates in decision tree analysis as verification indicators to try to pass different levels of verification. It also tries out different grade classification methods to see which one is the best; the analysis objects are divided into three categories: overall students, high-scoring students, and low-scoring students. And in this way, you can examine the learning habits of various students and draw some useful conclusions. Learning records are analyzed through the time dimension using the classification analysis method in data mining, and individual course decision-making rules are generated to give teachers a better understanding of the possible relationship between students' learning behaviors and learning outcomes. This article presents a comprehensive analysis algorithm based on intelligent information technology, and the decision rules generated can predict students' learning behavior to a degree. Teachers and experts who can verify and provide feedback on these decision rules will help them become more widely used teaching aids. It can be used in conjunction with CAI to provide teachers with more information about relevant learning records.

## Figures and Tables

**Table 1:** Average consistency index.

N	1	2	3	4	5	6	6	7	8	9	11
RI	0.00	0.02	0.056	0.91	1.11	1.23	1.32	1.44	1.48	1.52	1.54

## Data Availability

The data used to support the findings of this study are available from the corresponding author upon request.

## References

[1] Xu Z., Choo K. K. R., Dehghantanha A., Parizi R., Hammoudeh M. (2019). *Cyber Security Intelligence and Analytics*.

[2] Alomari M. A., Jabr M. O. (2020). The effect of the use of an educational software based on the strategy of artificial intelligence on students’ achievement and their attitudes towards it. *Management Science Letters*.

[3] CuI L. (2021). A preliminary study on the management strategy of university personnel files based on artificial intelligence technology. *Journal of Electronic Research and Application*.

[4] Zhou Z., Yu K., Economics S. O. (2019). Research on the teaching reform of college students’ mental health education under the strategy of “new economy and management”. *Journal of Heihe University*.

[5] Tan W. (2021). Research on the online interactive mode of “one village, one university student” program course: from an OBE perspective. *OALib*.

[6] Kaptein M. C., Markopoulos P., Ruyter B. D., Aarts E. (2011). Two acts of social intelligence. *AI & Society*.

[7] Zhou H., Lu J., Huang Y., Chen Y. (2021). Research on key technology of classroom teaching evaluation based on artificial intelligence. *Journal of Physics: Conference Series*.

[8] Luo X., Xie L. Research on Artificial Intelligence-Based Sharing Education in the Era of Internet+.

[9] Huang X., Zhao J., Fu J., Zhang X. (2020). Effectiveness of ideological and political education reform in universities based on data mining artificial intelligence technology. *Journal of Intelligent and Fuzzy Systems*.

[10] Kirichenko A. V., Reznichenko A. V., Ranepa Z. I. A. (2020). The methodology of applying the akmeologikal approach to education in a digital economy based on artificial intelligence. *Scholarly Journal Of Psychology And Behavioral Sciences*.

[11] Cui L. (2021). A preliminary study on the management strategy of university personnel files based on artificial intelligence technology. *Journal of Electronic Research and Application*.

[12] Jie H., Library J. (2018). Research on the application of artificial intelligence technology in information retrieval of public library. *Electronics Test*.

[13] Zhang Y. S., Xu Y. X., Fan W. L., Zhang Z. Y. (2019). Relationship between residual feed intake and production traits in a population of F_2_ d. *The Journal of Poultry Science*.

[14] Ting-Ting L. U. (2012). Research on overseas student management strategy in higher vocational colleges: taking changzhou college of information technology as an example. *Journal of Changzhou Vocational College of Information Technology*.

[15] Liu Y., University T. (2019). Research on strategy of university archives management under artificial intelligence environment. *Office Informatization*.

[16] Chen Y., Chen Y. (2021). Research on the application and influence of artificial intelligence technology. *Journal of Physics: Conference Series*.

[17] Zhang X., Chen L. (2021). College English smart classroom teaching model based on artificial intelligence technology in mobile information systems. *Mobile Information Systems*.

[18] Jin Q. H. (2018). Research on the possibility and development trend of artificial intelligence technology applied to music education. *Journal of Hubei Correspondence University*.

[19] Kordaki M., Daradoumis T., Fragidakis D., Grigoriadou M. (2012). Adapting the collaborative strategy ‘students team Achievement divisions’ in an information technology work place. *Intelligent Adaptation and Personalization Techniques in Computer-Supported Collaborative Learning*.

[20] Hu Y., Li Q. Q., Hsu S. W. (2021). Interactive visual computer vision analysis based on artificial intelligence technology in intelligent education. *Neural Computing & Applications*.

[21] Zhang Y. (2019). Research on precision teaching in artificial intelligence education model based on precision interactive behavior analysis of teaching. *Adult Education*.

[22] Liu Y. J., Rong H. U. (2019). Research on the information security situational awareness system based on big data and artificial intelligence technology. *Management & Technology of SME*.

[23] Yang N., Wang Y. (2012). Study on education resource search engine based on artificial intelligence technology[J]. *Journal of Jilin Institute of Architecture & Civil Engineering*.

[24] Lv X., Li M. (2021). Application and research of the intelligent management system based on Internet of Things technology in the era of big data. *Mobile Information Systems*.

